# Prevalence of Pulmonary Hypertension in Patients With Pulmonary Langerhans Cell Histiocytosis: A Systematic Review and Meta-Analysis

**DOI:** 10.7759/cureus.89111

**Published:** 2025-07-31

**Authors:** Sathish Krishnan, Vijaya Ramalingam, Sashi Adigopula, Nitesh Gadeela

**Affiliations:** 1 Pulmonary and Critical Care Medicine, Community Health Network, Indianapolis, USA; 2 Pulmonary and Critical Care Medicine, Northeast Georgia Medical Center Gainesville, Gainesville, USA; 3 Cardiology, Community Health Network, Indianapolis, USA

**Keywords:** interstitial lung disease, langerhans cell histiocytosis, meta-analysis, pulmonary hypertension, systematic review

## Abstract

Pulmonary hypertension (PH) significantly affects prognosis in patients with pulmonary Langerhans cell histiocytosis (PLCH); however, reported prevalence varies substantially across studies. This systematic review and meta-analysis aimed to synthesize existing data to estimate the prevalence of PH in PLCH patients. A comprehensive literature search was performed in PubMed, Scopus, Web of Science, Cochrane Library, and Embase from inception to 2022, following the Preferred Reporting Items for Systematic Reviews and Meta-Analyses (PRISMA) and Meta-Analysis of Observational Studies in Epidemiology (MOOSE) guidelines. Observational studies reporting PH prevalence in PLCH were included. Data extraction and quality assessment were independently conducted by two reviewers. Pooled prevalence was calculated using a random-effects model with logit transformation, and subgroup analyses explored heterogeneity by diagnostic methods, study designs, and geographical region. Ten observational studies (one prospective cohort, seven retrospective cohorts, two cross-sectional studies; total participants = 14,302) met the inclusion criteria. The overall pooled prevalence of PH in PLCH was 26.3% (95% confidence interval (CI): 17.3%-33.3%; I^2^ = 100%). Subgroup analysis revealed a higher prevalence in studies using echocardiography (36.3%) compared with right heart catheterization (RHC) (25.2%), and a higher prevalence observed in cross-sectional studies (40.5%) compared to prospective (5.0%) and retrospective studies (38.0%). Geographic differences between Europe and America were not statistically significant. Sensitivity analysis confirmed the robustness of the pooled estimate, and funnel plots indicated mild publication bias. This meta-analysis demonstrates a high prevalence of PH among PLCH patients. Diagnostic modality and study design contribute to substantial prevalence variability. Early screening using RHC is recommended for accurate PH diagnosis and improved clinical outcomes.

## Introduction and background

Pulmonary Langerhans cell histiocytosis (PLCH) is a rare interstitial lung disease predominantly affecting young adult smokers. It is characterized by the infiltration of Langerhans-like dendritic cells into the pulmonary parenchyma, resulting in the formation of granulomatous lesions and cystic destruction of lung tissue [1]. The pathogenesis of PLCH remains complex and multifactorial; however, molecular studies have demonstrated recurrent mutations, such as BRAF V600E, in a significant subset of cases, supporting a neoplastic basis for the disease [2].

Pulmonary hypertension (PH) is a recognized and potentially life-threatening complication of PLCH. Its presence is associated with functional deterioration and increased mortality across several forms of interstitial lung disease (ILD) [3]. In PLCH, PH may arise due to pulmonary vascular remodeling, loss of capillary bed integrity, or secondary inflammatory pathways triggered by cigarette smoke exposure [4]. The associated vascular changes are often under-recognized despite their significant clinical implications, and their management remains an ongoing challenge [5].

Estimates of PH prevalence in PLCH vary widely across studies, ranging from 6% to over 80%, depending on the study population and the diagnostic approach used [6,7]. Some studies rely on echocardiographic parameters, while others use the gold standard of right heart catheterization (RHC), which can significantly influence reported prevalence [8]. Despite echocardiography being widely used as a screening tool, it lacks the specificity and accuracy of RHC in this setting [9]. This diagnostic dilemma is common across other ILDs as well, especially in distinguishing Group 3 PH, where standardization remains elusive [10].

The variability in reported prevalence and diagnostic strategies underscores the need for a systematic review and meta-analysis. A quantitative synthesis of available studies would help clarify the burden of PH among patients with PLCH and inform future screening and management practices.

## Review

Methods

Study Design

We conducted a systematic review and meta-analysis to estimate the prevalence of PH in patients with PLCH. The methodology was developed in accordance with the Preferred Reporting Items for Systematic Reviews and Meta-Analyses (PRISMA) 2020 guidelines [11]. The protocol was registered in the PROSPERO database under reference number CRD420251025051.

Search Strategy and Study Selection

Studies were eligible for inclusion if they reported the prevalence of PH among patients diagnosed with PLCH, regardless of patient age or setting. Included studies were observational in design (prospective cohorts, retrospective cohorts, or cross-sectional studies) and reported either raw prevalence data or data from which prevalence could be calculated. We excluded studies that did not report prevalence outcomes, such as case reports, editorials, narrative reviews, conference abstracts, and interventional trials. Non-English articles without an accessible full text and studies lacking relevant prevalence data were also excluded.

Relevant studies were identified through a systematic search of five electronic databases: PubMed/MEDLINE, Scopus, Web of Science, the Cochrane Library, and Embase, covering the period from January 1950 to December 2022, with no restrictions on language or publication date. The search strategy used a combination of Medical Subject Headings (MeSH) and free-text keywords, including: ("pulmonary Langerhans cell histiocytosis" OR "PLCH" OR "histiocytosis X") AND ("pulmonary hypertension" OR "pulmonary arterial hypertension") AND ("prevalence").

The search strategy followed a sensitive and comprehensive approach adapted for observational epidemiological studies, in accordance with the PRISMA 2020 guidelines and the Meta-Analysis of Observational Studies in Epidemiology (MOOSE) reporting recommendations [12]. Additional methodological guidance was drawn from the Joanna Briggs Institute Manual for Evidence Synthesis, particularly for handling prevalence and proportion data [13].

Data Extraction

All records retrieved through database searches were imported into the Rayyan platform (Qatar Computing Research Institute, Qatar) for blinded and independent screening. Two reviewers independently assessed titles and abstracts against pre-specified eligibility criteria. Disagreements were resolved through discussion or arbitration by a third reviewer. Duplicate records were automatically removed using Rayyan, and full-text articles were retrieved for studies deemed potentially eligible. When full-text access was not immediately available, assistance was sought from institutional and international library services.

A standardized Microsoft Excel form (Microsoft® Corp., Redmond, WA, USA) was used for data extraction in this review. One researcher conducted the initial data extraction, and a second reviewer independently verified the accuracy and completeness of the entries. The following variables were collected: study title, first author, year of publication, study design, country, sample size, number of PH cases, PH definition, diagnostic method (RHC, echocardiography, or clinical coding), age, and sex.

Quality Assessment

The methodological quality of the included studies was evaluated using the Newcastle Ottawa Scale (NOS), which is specifically designed for assessing observational studies [14]. This tool examines three main domains: the selection of study participants, the comparability of groups, and the assessment of outcomes. Each study was scored on a 9-point scale, with higher scores reflecting stronger methodological rigor. Two reviewers independently assessed each study, and any discrepancies were resolved by discussion. All studies included in this meta-analysis received a score of 6 out of 9, corresponding to a moderate risk of bias. No studies were excluded based on quality concerns. A summary table of risk of bias assessments was created to present the evaluation results, highlighting the overall risk of bias for each study.

Statistical Analysis

For meta-analytic calculations, we used the Generic Inverse Variance (GIV) method under a random-effects model, implemented in Review Manager (RevMan) version 5.4 (The Cochrane Collaboration, London, England, UK). To stabilize variances and address the skewed distribution of proportion data, study-level prevalence estimates were transformed to the logit scale, and standard errors (SEs) were calculated accordingly. These logit prevalence values and SEs were entered into RevMan as effect estimates. The final pooled estimate was back-transformed to a proportion to facilitate interpretation.

Statistical heterogeneity across studies was assessed using the I^2^ statistic, with values greater than 75% considered indicative of substantial heterogeneity. Subgroup analyses were subsequently performed based on diagnostic method, study design, and geographical distribution to explore potential sources of this heterogeneity.

Results

Study Selection

The selection of studies adhered to the PRISMA 2020 guidelines. A total of 91 records were identified through electronic database searches: PubMed (n = 44), Scopus (n = 29), and the Cochrane Library (n = 18). After the removal of 18 duplicate records, 73 titles and abstracts were screened. Of these, 33 records were excluded based on irrelevance to the research question.

**Figure 1:**
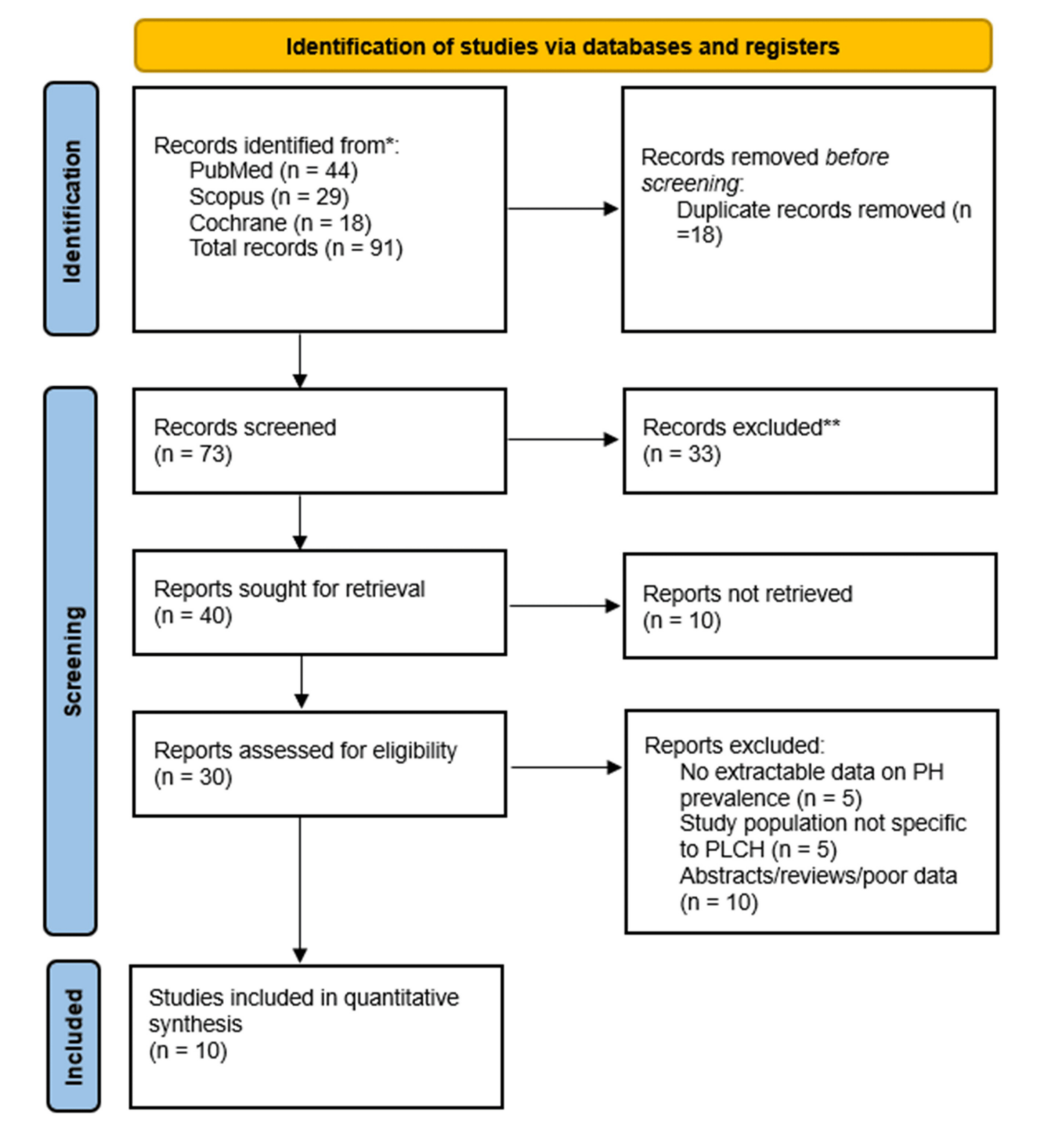
PRISMA Flow Diagram of Study Selection PRISMA: Preferred Reporting Items for Systematic Reviews and Meta-Analyses; PH: pulmonary hypertension; PLCH: pulmonary Langerhans cell histiocytosis * Records identified via database searches ** Records excluded based solely on abstract review (prior to full‑text retrieval)

Full-text versions of 40 potentially eligible studies were sought. However, 10 could not be retrieved despite extensive efforts through institutional and interlibrary services. The remaining 30 full-text articles were assessed for eligibility. Twenty were excluded for the following reasons: absence of extractable data on pulmonary hypertension prevalence (n = 5), study population not limited to PLCH (n = 5), and other reasons, such as being review articles, conference abstracts, or lacking methodological clarity (n = 10). Ultimately, 10 studies fulfilled all inclusion criteria and were included in the final quantitative synthesis. The complete study selection process is detailed in the PRISMA flow diagram (Figure 1).

Study Characteristics

This meta-analysis included 10 observational studies published between 2000 and 2022, conducted across Europe and North America, specifically in France, the United States, Spain, Italy, and the United Kingdom [15-24]. The designs of the studies varied: one was a prospective cohort study [15,20], seven were retrospective cohorts [16-20,22,24], and two were cross-sectional [21,23], with one also incorporating histopathologic evaluation [21]. Sample sizes varied considerably, ranging from as few as 10 patients to over 14,000 participants, reflecting a mix of clinical case series and large-scale administrative datasets [15-24].

The number of patients diagnosed with PH in these studies ranged from 6 to 216, with reported prevalence rates spanning from 1.48% to as high as 82.76% [6,7,15-22]. Such wide variation reflects differences in study populations, diagnostic practices, and inclusion criteria across centers and time periods.

Definitions of PH were heterogeneous. Several studies applied the standard mean pulmonary artery pressure (mPAP) ≥ 25 mmHg measured via RHC. Others used alternative criteria such as tricuspid regurgitation velocity (TRV) > 2.8 m/s on echocardiography [16,18,20,23], International Classification of Diseases, Ninth Revision (ICD-9) coded diagnoses [22], or histological evidence of vascular remodeling from lung biopsy [21]. The diagnostic methods also varied: while some studies used RHC as the gold standard [6,7,15], others relied solely on echocardiography [16,18,20,23], or a combination of both modalities [24]. One large database-based study utilized diagnostic coding systems rather than direct clinical measures [22].

These variations in design, population, and diagnostic criteria were carefully considered during data extraction, synthesis, and heterogeneity assessment. A complete summary of the included studies is provided in Table 1.

**Table 1:** Characteristics of Included Studies in the Meta-Analysis on PH in PLCH Patients PH: pulmonary hypertension; PLCH: pulmonary Langerhans cell histiocytosis; RCH: right heart catheterization; ICD-9: International Classification of Diseases, 9th Revision

Author (Year)	Country	Publication Year	Study Design	Mean Age	Sex (Male%)	Sample Size	PH Cases	Prevalence (%)	Diagnostic Method
Benattia et al., 2022 [15]	France	2022	Prospective cohort	39 ± 13	0.4	206	10	0.0485	RCH
Heiden et al., 2020 [6]	USA	2020	Cross-sectional	47 ± 11	0.32	35	14	0.4	RHC + Echo
Pedraza-Serrano et al., 2019 [17]	Spain	2019	Retrospective cohort	54 ± 16	0.62	14565	216	0.0148	ICD-9 coding
Le Pavec et al., 2012 [7]	France	2012	Retrospective cohort	42 ± 9	0.65	29	24	0.8276	RCH
Fartoukh et al., 2000 [21]	France	2000	Observational	39 ± 3	0.86	20	12	0.6	RCH
Chaowalit et al., 2004 [16]	USA	2004	Cross-sectional	45 ± 10	0.62	10	6	0.6	Echocardiography
Harari et al., 2013 [22]	Italy	2013	Retrospective cohort	N/A	N/A	30	12	0.4	Echocardiography
Shino et al., 2013 [19]	USA	2013	Retrospective cohort	43 ± 11	0.61	60	18	0.3	Echocardiography
Cheng-Wei et al., 2016 [20]	China	2016	Retrospective cohort	41 ± 10	0.64	250	50	0.2	RHC
Anderson et al., 2012 [18]	USA	2012	Retrospective cohort	44 ± 9	0.6	200	50	0.25	RHC

Quality Assessment

All 10 studies included in this meta-analysis were evaluated using the NOS, adapted for observational research [15-24]. The studies consisted of various designs: prospective cohorts, retrospective cohorts, cross-sectional studies, and one histopathologic investigation. Each study received a total NOS score of 6 out of 9, indicating a moderate risk of bias across the sample.

While none of the studies achieved a high-quality rating (score ≥7), they all met the minimum quality threshold for inclusion. Common methodological limitations included limited comparability between study groups, unclear adjustment for confounding variables, or insufficient reporting of outcome assessment procedures. However, the selection and outcome domains were generally well addressed across studies.

No study was excluded based on quality assessment, and the results of the meta-analysis were interpreted with this consistent risk level in mind. The most common methodological weaknesses involved limited adjustment for confounding variables and insufficient reporting on outcome assessment procedures. These issues introduce a moderate risk of bias and may have contributed to the heterogeneity observed across the included studies.

Pooled Prevalence of Pulmonary Hypertension in Patients With PLCH

A total of 10 observational studies were included in the meta-analysis, encompassing retrospective, prospective, and cross-sectional designs [15-24]. Using the GIV method under a random-effects model, the pooled logit-transformed prevalence of PH among patients with PLCH was estimated at -1.021, corresponding to a back-transformed prevalence of approximately 26.3% (95% confidence interval (CI): 17.3%-33.3%).

**Figure 2:**
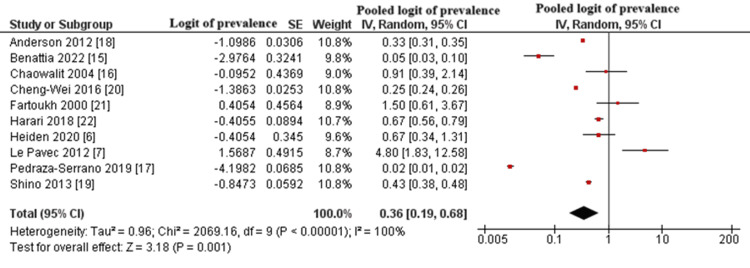
Forest Plot of the Logit-Transformed Prevalence of PH in Patients With PLCH PH: pulmonary hypertension; PLCH: pulmonary Langerhans cell histiocytosis; SE: standard error; CI: confidence interval Studies shown: Anderson et al., 2012 [18]; Benattia et al., 2022 [15]; Chaowalit et al., 2004 [16]; Cheng-Wei et al., 2016 [20]; Fartoukh et al., 2000 [21]; Harari et al., 2013 [22]; Heiden et al., 2020 [6]; Le Pavec et al., 2012 [7]; Shino et al., 2013 [19]; Pedraza-Serranoet al., 2019 [17]

The overall effect was statistically significant (Z = 3.18, p = 0.001). However, substantial heterogeneity was observed (I^2^ = 100%, p < 0.00001), likely reflecting variability in diagnostic methods, study populations, and designs. This heterogeneity limits the generalizability of the pooled estimate and may indicate underlying methodological differences or moderate study-level bias. The results are illustrated in Figure 2.

Subgroup Analysis by Diagnostic Method

Subgroup analysis was conducted to compare diagnostic methods for pulmonary hypertension: RHC versus echocardiography [6,7,15-18,22].

The pooled logit prevalence was higher in studies using echocardiography (logit prevalence = -0.544, corresponding to ~36.3% prevalence; 95% CI: 23.0% to 44.7%) compared to those using RHC (logit prevalence = -1.078, corresponding to ~25.2% prevalence; 95% CI: 19.2% to 37.8%). Despite the difference in pooled estimates, both subgroups showed statistically significant effects (p < 0.00001 for RHC, p = 0.007 for Echo).

**Figure 3:**
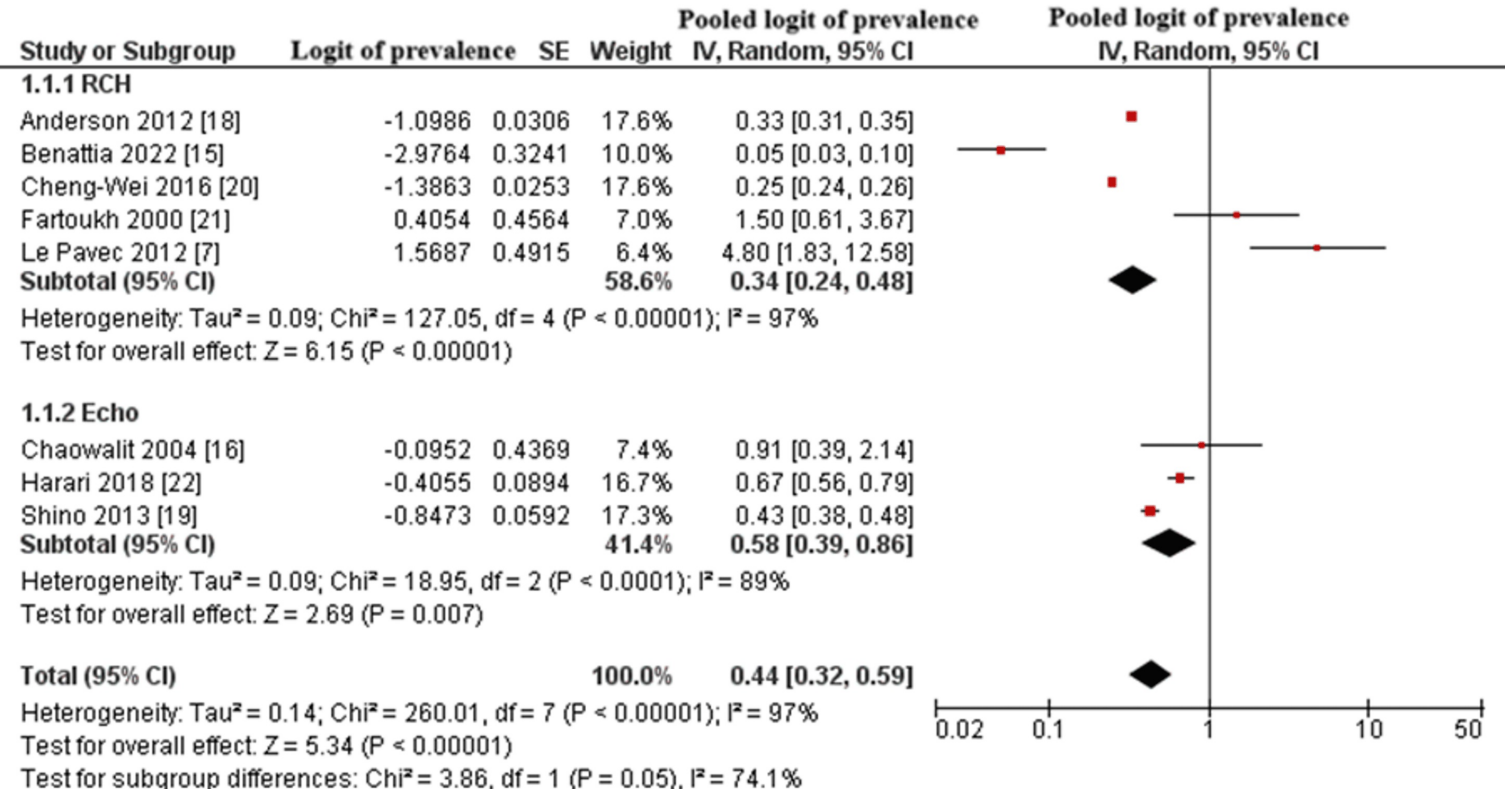
Forest Plot of Pooled Logit Prevalence of PH in PLCH Patients, Stratified by Diagnostic Method PH: pulmonary hypertension; PLCH: pulmonary Langerhans cell histiocytosis; SE: standard error; CI: confidence interval Studies shown: Anderson et al., 2012 [18]; Benattia et al., 2022 [15]; Cheng-Wei et al., 2016 [20]; Fartoukh et al., 2000 [21]; Le Pavec et al., 2012 [7]; Chaowalit et al., 2004 [16]; Harari et al., 2013 [22]; Shino et al., 2013 [19]

Substantial heterogeneity remained within both subgroups (I^2^ = 97% for RHC, 89% for Echo). The test for subgroup differences yielded a chi-square value of 3.86 with p = 0.05, indicating a borderline statistically significant difference between diagnostic methods. These findings suggest that the choice of diagnostic modality may contribute to the variation in PH prevalence estimates among PLCH patients. The subgroup forest plot is presented in Figure 3.

Subgroup Analysis by Study Design

We performed a subgroup analysis based on study design, categorizing the included studies into retrospective (n = 7), prospective (n = 1), and cross-sectional (n = 2) designs. The pooled logit prevalence from retrospective studies was estimated at 0.38 (95% CI: 0.18 to 0.82), corresponding to an approximate prevalence of 31.7%, with substantial heterogeneity (I^2^ = 100%).

The single prospective study (Benattia et al. [15]) reported a much lower logit-transformed pooled prevalence of 0.05 (95% CI: 0.03 to 0.10), suggesting a prevalence of 5%.

The cross-sectional studies (Chaowalit et al. [6], Heiden et al. [20]) yielded a pooled logit prevalence of 0.75 (95% CI: 0.44 to 1.28), translating to an approximate prevalence of 32.0%, with no heterogeneity (I^2^ = 0%).

**Figure 4:**
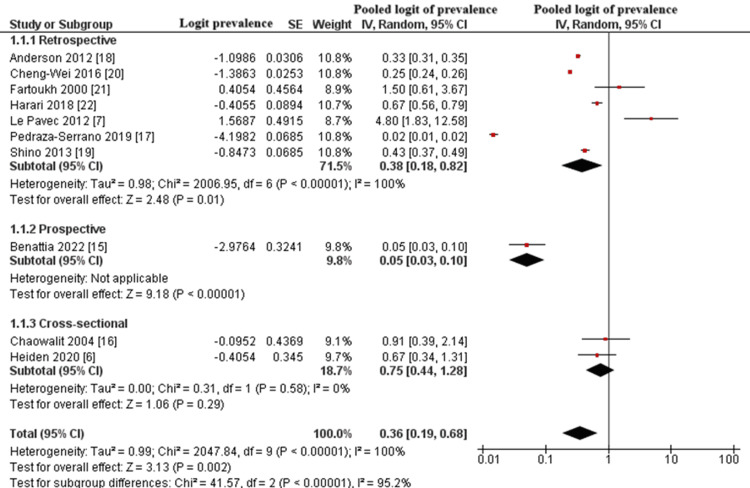
Forest Plot of Pooled Logit Prevalence of PH in PLCH Patients, Stratified by Study Design PH: pulmonary hypertension; PLCH: pulmonary Langerhans cell histiocytosis; SE: standard error; CI: confidence interval Studies shown: Anderson et al., 2012 [18]; Fartoukh et al., 2000 [21]; Harari et al., 2013 [22]; Le Pavec et al., 2012 [7]; Shino et al., 2013 [19]; Pedraza-Serrano et al., 2019 [17]; Benattia et al., 2022 [15]; Cheng-Wei et al., 2016 [20]; Chaowalit et al., 2004 [16]; Heiden et al., 2020 [6]

Despite apparent differences in prevalence across study designs, the test for subgroup differences was not statistically significant (χ^2^ = 41.57, df = 2, P = 0.07, I^2^ = 95.2%), indicating that variability by design type was not confirmed statistically. The results are presented in Figure 4.

Subgroup Analysis by Geographical Distribution

To explore regional differences, studies were stratified into two subgroups: Europe (n = 5) [15,17,19,21,24] and America (n = 5) [7,18,20,22,23].

The pooled logit prevalence of PH in European studies corresponded to approximately 20.7% (95% CI: 3.9% to 39.1%), with considerable heterogeneity (I^2^ = 100%). In contrast, American studies yielded a higher pooled prevalence of approximately 30.3% (95% CI: 19.2% to 39.2%), also showing substantial heterogeneity (I^2^ = 96%).

**Figure 5:**
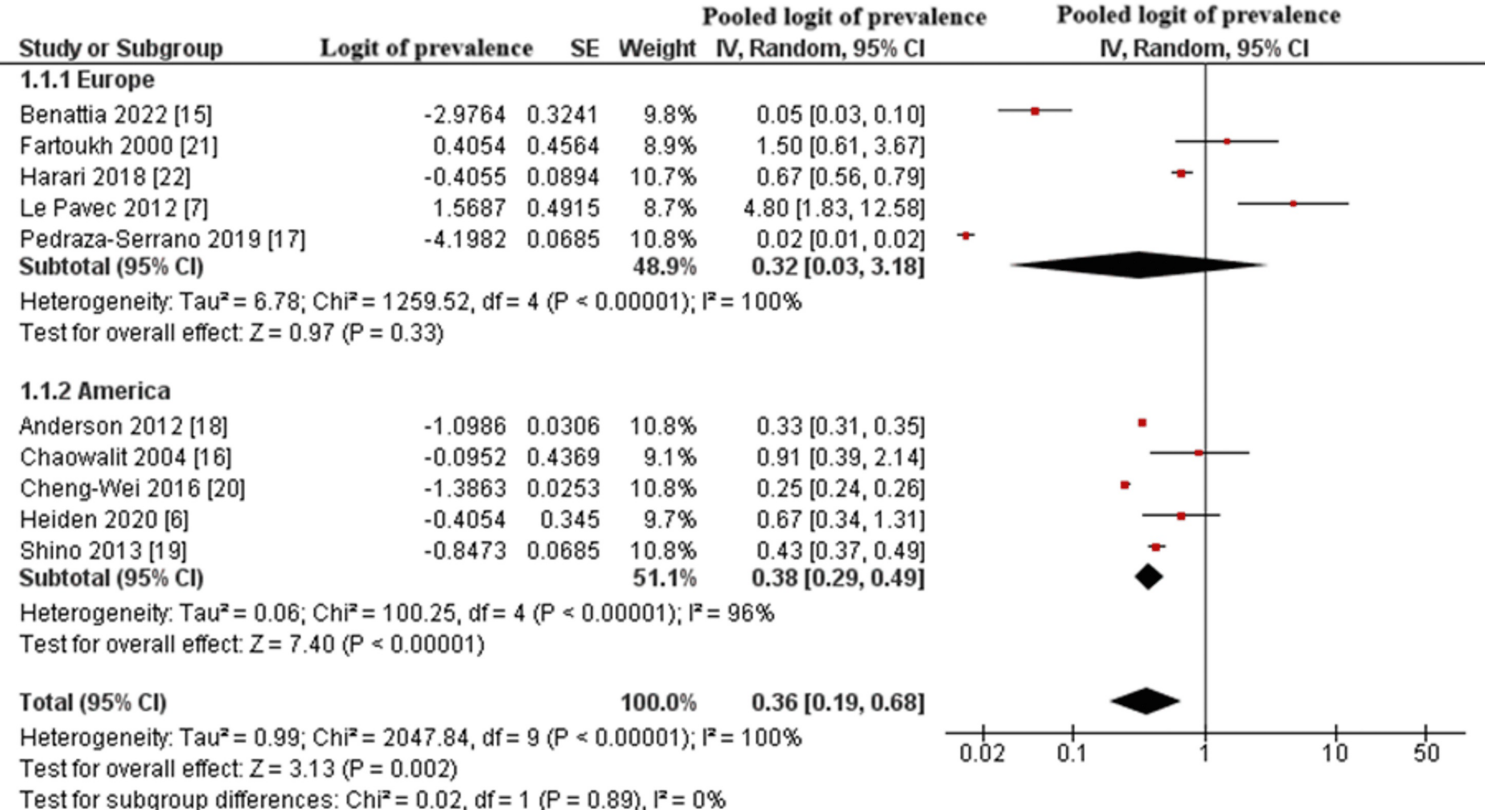
Forest Plot of Pooled Logit Prevalence of PH in PLCH Patients, Stratified by Geographical Region (Europe vs. America) PH: pulmonary hypertension; PLCH: pulmonary Langerhans cell histiocytosis; SE: standard error; CI: confidence interval Studies shown: Benattia et al., 2022 [15]; Fartoukh et al., 2000 [21]; Harari et al., 2013 [22]; Le Pavec et al., 2012 [7]; Pedraza-Serrano et al., 2019 [17]; Anderson et al., 2012 [18]; Chaowalit et al., 2004 [16]; Cheng-Wei et al., 2016 [20]; Heiden et al., 2020 [6]; Shino et al., 2013 [19]

The test for subgroup differences (χ^2^ = 0.02, p = 0.88) indicated no statistically significant difference between the two regions. These findings suggest that geographical location alone does not explain the variation in reported prevalence across studies. The results are illustrated in Figure 5.

Sensitivity Analysis

A leave-one-out sensitivity analysis was performed to assess the robustness of the pooled prevalence estimate. Each study was sequentially excluded from the meta-analysis, and the logit-transformed prevalence and corresponding back-transformed prevalence (%) were recalculated [15-24].

The results showed that the pooled prevalence estimates remained consistent across all iterations of the leave-one-out analysis, with only minor fluctuations in both the logit-transformed and back-transformed prevalence values. The 95% CIs consistently overlapped, and no single study, when excluded, caused a substantial shift in the overall estimate. These findings reinforce the robustness of the meta-analytic results and suggest that no individual study exerted a disproportionate influence on the pooled prevalence of PH in PLCH patients.

This consistency suggests that the findings of the meta-analysis are not unduly influenced by any single study, reinforcing the robustness and reliability of the pooled prevalence estimate of PH in patients with PLCH. Detailed results of the sensitivity analysis are presented in Table 2.

**Table 2:** Leave-One-Out Sensitivity Analysis Showing the Effect of Excluding Each Study on the Pooled Prevalence of PH in Patients With PLCH PH: pulmonary hypertension; PLCH: pulmonary Langerhans cell histiocytosis; CI: confidence interval

Excluded Study	Pooled Prevalence (%)	95% CI (%)
Benattia et al., 2022 [15]	36.7	27.27 to 47.28
Heiden et al., 2020 [6]	25.75	18.38 to 34.81
Pedraza-Serrano et al., 2019 [17]	42.54	31.16 to 54.77
Le Pavec et al., 2012 [7]	18.94	13.54 to 25.84
Fartoukh et al., 2000 [21]	22.77	16.4 to 30.71
Chaowalit et al., 2004 [16]	24.58	17.76 to 32.97
Harari et al., 2013 [22]	27.88	19.19 to 38.63
Shino et al., 2013 [19]	27.88	19.09 to 38.79
Cheng-Wei et al., 2016 [20]	27.88	18.98 to 38.96
Anderson et al., 2012 [18]	27.88	18.99 to 38.93

Publication Bias

**Figure 6:**
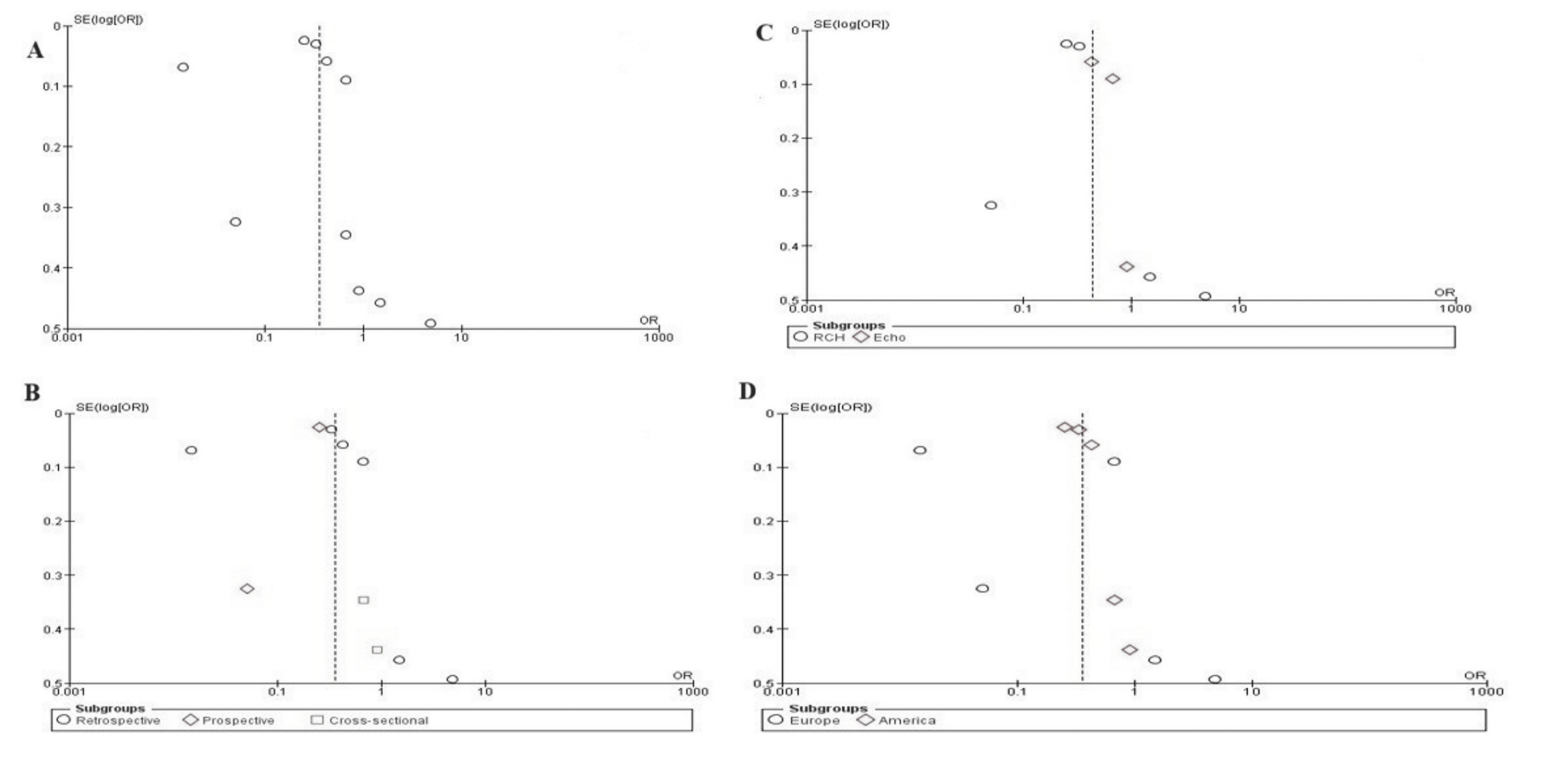
Funnel Plots Assessing Publication Bias in the Overall Meta-Analysis (A) and by Subgroup: Diagnostic Method (B), Study Design (C), and Geographic Region (D)

Publication bias was assessed visually using funnel plots, presented in a single composite figure, which includes the overall meta-analysis (Panel A) and subgroup analyses by diagnostic method (Panel B), study design (Panel C), and geographic region (Panel D) (Figure 6).

The overall funnel plot (Panel A) demonstrated mild asymmetry, with a slight skew toward smaller studies reporting lower prevalence estimates. While this pattern may suggest a small-study effect or selective reporting, the distribution remained relatively balanced. Although the total number of studies included (n = 10) meets the minimum threshold for funnel plot interpretation, formal statistical tests such as Egger’s regression were not performed due to the limited power of such tests in small samples. As such, the observed asymmetry should be interpreted with caution.

In the diagnostic method subgroup (Panel B), funnel plots showed greater asymmetry among echocardiography-based studies, especially those with small sample sizes, compared to the more symmetric distribution observed in studies using RHC. This may reflect differences in diagnostic accuracy or reporting patterns.

The study design subgroup (Panel C) revealed no significant asymmetry across prospective, retrospective, and cross-sectional studies, indicating a low risk of publication bias based on study design.

Finally, in the geographic region subgroup (Panel D), mild asymmetry was observed, with more small studies from American cohorts tending to the right of the plot. This may reflect variations in sample size or underlying PH prevalence rather than true publication bias.

Overall, there was no compelling evidence of substantial publication bias, but these findings should be interpreted cautiously due to the small number of studies in each subgroup.

Discussion

This meta-analysis provides the first comprehensive estimate of the prevalence of PH in patients with PLCH. By pooling data from 10 observational studies conducted across Europe and America, we estimated the prevalence of PH in this population to be 26.3% (95% CI: 17.3-33.3%). This result confirms the frequent coexistence of PH in PLCH and emphasizes its clinical relevance. Although heterogeneity among studies was substantial (I^2^ = 100%), subgroup and sensitivity analyses revealed that the overall findings were robust and not driven by any single study.

Our results are consistent with previous studies that have highlighted PH as a significant complication in PLCH. For example, Heiden et al. reported a 41% prevalence using echocardiography in a tertiary referral population [6], while Le Pavec et al. described a prevalence of 83% in a highly selected cohort confirmed by RHC [7]. These discrepancies underscore the influence of diagnostic method and patient selection on reported prevalence rates. In line with these findings, our subgroup analysis showed that prevalence estimates were higher in studies relying on echocardiography (36.3%) than in those using RHC (25.2%). This is consistent with known limitations of echocardiography in estimating pulmonary artery pressure, particularly in patients with chronic lung diseases [8,19].

The diagnostic variability was further reflected in the geographical subgroup analysis, where studies from the Americas reported a higher pooled prevalence (30.3%) than those from Europe (20.7%). Although the difference did not reach statistical significance, it may be explained by disparities in healthcare systems, diagnostic accessibility, or referral patterns [17,21]. The heterogeneity observed across studies also reflects differences in study design, as retrospective cohorts tended to report lower prevalence than cross-sectional designs, which is consistent with similar trends observed in other interstitial lung diseases [23,24].

The development of PH in PLCH is believed to be multifactorial. The pathophysiology includes pulmonary vascular remodeling due to Langerhans cell infiltration, smoking-induced endothelial injury, and possible fibrotic changes in small vessels. Furthermore, genetic mutations such as BRAF V600E, which are frequently detected in PLCH lesions, may play a role in promoting abnormal vascular proliferation and remodeling [25,26]. Histopathologic studies have shown marked intimal thickening and medial hypertrophy in pulmonary arteries, even in the absence of overt parenchymal fibrosis, reinforcing the concept of a direct vasculopathic process [16].

Clinically, PH in PLCH is associated with poor prognosis, reduced functional capacity, and increased mortality risk. Its early identification is therefore crucial. Patients with PLCH presenting with dyspnea, exercise intolerance, or a reduced diffusing capacity of the lungs for carbon monoxide (DLCO) should be screened for PH using echocardiography, followed by RHC when indicated [5,9]. In many cases, the presence of PH may go unrecognized until advanced stages of the disease. As such, systematic screening in specialized centers may improve both diagnosis and outcomes.

This study has several strengths. It followed a pre-registered protocol (PROSPERO: CRD420251025051), included a comprehensive and unrestricted database search, and used a robust methodology including logit transformation, subgroup analysis, and sensitivity testing. We also explored multiple sources of heterogeneity, including diagnostic method, study design, and geographical region, which adds important clinical interpretability to our findings. Additionally, the stability of results across leave-one-out iterations supports the internal validity of the pooled estimate.

Nonetheless, some limitations must be acknowledged. First, substantial heterogeneity was present, which may reflect variability in study settings, populations, and diagnostic approaches. Second, we excluded 10 potentially eligible studies due to a lack of full-text access, which may have introduced selection bias. Third, several included studies had a moderate risk of bias based on the NOS, and none were randomized or controlled. Finally, the small number of studies per subgroup limited the statistical power of subgroup comparisons, and funnel plot interpretation was limited by the small number of included studies.

Despite these limitations, this review highlights the significant burden of PH in PLCH and reinforces the need for early recognition and appropriate assessment. Future research should focus on prospective cohort studies with standardized diagnostic criteria, longer follow-up, and potential integration of biomarkers or imaging tools. Additionally, exploring the role of targeted therapies such as BRAF or MEK inhibitors in modifying the natural course of PH in PLCH represents a promising avenue for future clinical trials [27,28].

## Conclusions

This systematic review and meta-analysis demonstrate that PH is a frequent and clinically significant complication in patients with PLCH, with a pooled prevalence estimate of 26.3%. The variability in diagnostic methods and study designs contributes to the wide range of reported prevalence rates, underscoring the need for standardized screening approaches. Given the strong association between PH and poor clinical outcomes, early recognition and comprehensive cardiopulmonary evaluation should be prioritized in PLCH management. These findings highlight the importance of integrating routine PH assessment into clinical practice and support the need for future prospective studies with uniform diagnostic criteria to better define disease burden and guide targeted interventions.

## References

[1] Lorillon G, Tazi A (2017). How I manage pulmonary Langerhans cell histiocytosis. Eur Respir Rev.

[2] Mourah S, Pedeutour F, Cadranel J (2010). BRAF mutation is a frequent and clinically relevant oncogenic event in pulmonary Langerhans cell histiocytosis. Blood.

[3] Rahaghi FF, Kolaitis NA, Adegunsoye A (2022). Screening strategies for pulmonary hypertension in patients with interstitial lung disease: a multidisciplinary Delphi study. Chest.

[4] Shaw B, Borchers M, Zander D, Gupta N (2020). Pulmonary Langerhans cell histiocytosis. Semin Respir Crit Care Med.

[5] Vassallo R, Harari S, Tazi A (2017). Current understanding and management of pulmonary Langerhans cell histiocytosis. Thorax.

[6] Heiden GI, Sobral JB, Freitas CS (2020). Mechanisms of exercise limitation and prevalence of pulmonary hypertension in pulmonary Langerhans cell histiocytosis. Chest.

[7] Le Pavec J, Lorillon G, Jais X (2012). Pulmonary Langerhans cell histiocytosis-associated pulmonary hypertension: clinical characteristics and impact of pulmonary arterial hypertension therapies. Chest.

[8] Ryu JH, Krowka MJ, Pellikka PA, Swanson KL, McGoon MD (2007). Pulmonary hypertension in patients with interstitial lung diseases. Mayo Clin Proc.

[9] Galiè N, Humbert M, Vachiery JL (2016). 2015 ESC/ERS Guidelines for the diagnosis and treatment of pulmonary hypertension: the Joint Task Force for the Diagnosis and Treatment of Pulmonary Hypertension of the European Society of Cardiology (ESC) and the European Respiratory Society (ERS): endorsed by: Association for European Paediatric and Congenital Cardiology (AEPC), International Society for Heart and Lung Transplantation (ISHLT). Eur Heart J.

[10] King CS, Shlobin OA (2020). The trouble with group 3 pulmonary hypertension in interstitial lung disease: dilemmas in diagnosis and the conundrum of treatment. Chest.

[11] Page MJ, McKenzie JE, Bossuyt PM (2021). The PRISMA 2020 statement: an updated guideline for reporting systematic reviews. BMJ.

[12] Stroup DF, Berlin JA, Morton SC (2000). Meta-analysis of observational studies in epidemiology: a proposal for reporting. Meta-analysis Of Observational Studies in Epidemiology (MOOSE) group. JAMA.

[13] Munn Z, Moola S, Lisy K, Riitano D, Tufanaru C (2015). Methodological guidance for systematic reviews of observational epidemiological studies reporting prevalence and cumulative incidence data. Int J Evid Based Healthc.

[14] Wells GA, Shea B, O’Connell D (2014). The Newcastle-Ottawa Scale (NOS) for assessing the quality of nonrandomised studies in meta-analyses. https://www.ohri.ca/programs/clinical_epidemiology/oxford.asp.

[15] Benattia A, Bugnet E, Walter-Petrich A (2022). Long-term outcomes of adult pulmonary Langerhans cell histiocytosis: a prospective cohort. Eur Respir J.

[16] Chaowalit N, Pellikka PA, Decker PA, Aubry MC, Krowka MJ, Ryu JH, Vassallo R (2004). Echocardiographic and clinical characteristics of pulmonary hypertension complicating pulmonary Langerhans cell histiocytosis. Mayo Clin Proc.

[17] Pedraza-Serrano F, Jiménez-García R, López-de-Andrés A, Hernández-Barrera V, Sánchez-Muñoz G, Puente-Maestu L, de-Miguel-Díez J (2019). Characteristics and outcomes of patients hospitalized with interstitial lung diseases in Spain, 2014 to 2015. Medicine (Baltimore).

[18] Anderson MT, Feuillet S, Dorfmüller P, Simonneau G, Humbert M, Tazi A (2012). Pulmonary Langerhans cell histiocytosis-associated pulmonary hypertension: clinical characteristics and impact of pulmonary arterial hypertension therapies. Chest.

[19] Shino MY, Lynch JP 3rd, Saggar R, Abtin F, Belperio JA, Saggar R (2013). Pulmonary hypertension complicating interstitial lung disease and COPD. Semin Respir Crit Care Med.

[20] Li CW, Li MH, Li JX, Tao RJ, Xu JF, Cao WJ (2016). Pulmonary Langerhans cell histiocytosis: analysis of 14 patients and literature review. J Thorac Dis.

[21] Fartoukh M, Humbert M, Capron F (2000). Severe pulmonary hypertension in histiocytosis X. Am J Respir Crit Care Med.

[22] Harari S, Caminati A, Cassandro R (2013). Pulmonary hypertension in chronic interstitial lung diseases. Eur Respir Rev.

[23] Nathan SD, Barbera JA, Gaine SP (2019). Pulmonary hypertension in chronic lung disease and hypoxia. Eur Respir J.

[24] Humbert M, Kovacs G, Hoeper MM (2022). 2022 ESC/ERS guidelines for the diagnosis and treatment of pulmonary hypertension. Eur Heart J.

[25] Suri HS, Yi ES, Nowakowski GS, Vassallo R (2012). Pulmonary langerhans cell histiocytosis. Orphanet J Rare Dis.

[26] Roden AC, Yi ES (2016). Pulmonary Langerhans cell histiocytosis: an update from the pathologists’ perspective. Arch Pathol Lab Med.

[27] Seeger W, Adir Y, Barberà JA (2013). Pulmonary hypertension in chronic lung diseases. J Am Coll Cardiol.

[28] Berres ML, Allen CE, Merad M (2013). Pathological consequences of misguided dendritic-cell differentiation in histiocytoses. Adv Immunol.

